# Vocalizations of female frogs contain nonlinear characteristics and individual signatures

**DOI:** 10.1371/journal.pone.0174815

**Published:** 2017-03-30

**Authors:** Fang Zhang, Juan Zhao, Albert S. Feng

**Affiliations:** 1 College of Life Sciences, Anhui Normal University, Wuhu, Anhui, China; 2 Department of Molecular and Integrative Physiology & Beckman Institute, University of Illinois at Urbana-Champaign, Urbana, IL, United States of America; University of Pavia, ITALY

## Abstract

Anuran vocalization is sexually dimorphic, with males doing the bulk of vocalizing. Female vocalization is rare and has been observed in a handful of species, including the concave-eared torrent frog (*Odorrana tormota*). Females *O*. *tormota* have been reported to emit moderate-level calls to attract males. In contrast to males, female’s vocal signals show no evidence of nonlinear phenomena (NLP). However, with females emitting calls so infrequently that this conclusion must be considered tentative in light of the limited supporting data. The present study was undertaken to test the hypotheses that their vocalizations: 1. may not be purely linear, 2. may contain individual signatures, similar to their male counterparts. We recorded 671 calls from six captive gravid females and found that their vocalizations are as complex as male calls, with numerous calls exhibiting complex upward/downward frequency modulations, and 39% of female calls containing at least one component of the NLP, i.e., subharmonics, deterministic chaos, frequency jump, or biphonation. Furthermore, females in captivity tend to call in bouts throughout the day and night, and the call rate varies hourly with a maximum of >10 calls per minute matching the maximum call rate in males. Similar to males, female vocalizations carry individual signatures, and all sound parameters analyzed differ significantly between individuals. This represents the first report ever showing that vocalizations of female anurans: 1. contain NLP, 2. carry individual signatures. Presence of signatures in both the male and female vocalizations opens up the possibility for males (and females) to distinguish individual frogs in both sexes acoustically, and thus their sound communication ability may be more advanced than previously thought.

## Introduction

Male concave-eared torrent frogs (*Odorrana tormota*) represent one of two anuran species known to communicate by ultrasounds [1–2]. Their vocal signals are diverse and complex, displaying multiple higher harmonics that extend well into the ultrasonic range and prominent nonlinear phenomena (NLP), such as subharmonics, deterministic chaos, frequency jump, and biphonation [3–4]. Females have been shown to also emit courtship calls during the reproductive season and their vocal signals were reportedly to be distinct from those of the male counterparts [5]. Further, they call infrequently, and their vocal signals do not exhibit NLP and have higher fundamental frequency compared to male's vocalizations [5]. As the vocalization data set used to derive these characterizations were very limited, it is unclear whether the depictions can be generalized to all females.

NLP has never been shown in female vocalizations for any anuran species. In male *O*. *tormota*, the vocal folds are structurally complex and the complexity is believed to contribute to the occurrence of NLP [6]. Similarly, females also possess complex vocal folds, albeit their vocal folds are bigger and more massive [6]. In light of the structural similarities of the vocal folds in the two sexes, we posit that females might have the capacity to emit calls containing NLP. A goal of the present study was to test this hypothesis.

Male *O*. *tormota* exhibit individual-specific call characteristics [4] which enable them to discriminate strangers from neighbors acoustically [7]. Vocal signatures have also been shown for males of several anuran species [8–11]. To date, there is no evidence for vocal signatures for anuran females. Female vocalization itself is rare to begin with, and has only been reported for a handful of species [5,12–19]. Given that female *O*. *tormota* show overt differential body size and thus their vocal apparati likely have differential size and/or shape which may contribute to individual specificity in their vocalizations. The second goal of this study was to investigate whether or not female calls contain individual-specific characteristics.

## Materials and methods

### Study site

Field study was performed in the mountain range of Huangshan (Anhui Province, China) in the village Fuxi (118°8’44.89”, 30°5’1.61”N, Elevation: 600 m), along Fu Creek, in the month of April, 2013 and from April 2 to May 3, 2015. The nightly ambient temperature at the study site during the study periods fluctuated widely but the temperature and humidity at which females were spotted in the wild fell within limited ranges, from 16.0° to 22.5°C and from 74 to 100%, respectively.

Seven gravid females, along with 28 solo non-gravid females, were captured by hand during the two study periods. The gravid females were all caught as amplexed pairs; the male amplexus partners were immediately removed upon capture. Females’ snout-vent lengths were measured with a fine caliper, and then housed individually in plastic cages (22 cm: 32 cm: 17 cm W:L:H) and placed indoors for a period of 36–48 hours. Mealworms were provided *ad libitum* under human care. The temperature and humidity indoors ranged from 16 to 19°C and 80 to 100%, respectively (measured during the sound recording sessions). The noise level indoors was measured using an SPL meter (TES 1357; Tianjin Sheng Xing Tai Technology Co., Tianjin, China) and it ranged between 38 and 43 dB SPL RMS–this was substantially lower than that of the ambient noise level outdoors along Fu Creek (range: 62–78 dB SPL; [20]).

### Sound recordings

Recording of female vocalizations was made using a digital audio recorder (Sound Devices 702, Sound Devices, WI, USA; frequency range: 10 Hz–96 kHz) and a miniature omni-directional condenser microphone (AKG model C417, AKG Acoustics, Vienna, Austria) with a flat frequency response over 20–20,000 Hz and a drop of a mere 10 dB at 30,000 Hz, using a sampling rate of 96 kHz and 16-bit accuracy. The microphone was mounted on a tripod and placed above the plastic cage at a height of 20 cm from the floor of the cage. Recordings were carried out mainly at evening hours between 20:00 and 24:00 hours in 2013 (N = 2), and throughout the day and night in 2015 (N = 5). At the conclusion of the recording sessions, the females were paired with males to form amplexus pairs again before they were released to the wild.

The experimental protocol was reviewed and approved by the Institutional Animal Care and Use Committees at the Anhui Normal University (IACUC #00111). No specific permissions were required for our study sites/activities, and the field studies did not involve endangered or protected species.

### Call analysis

**Fig 1:**
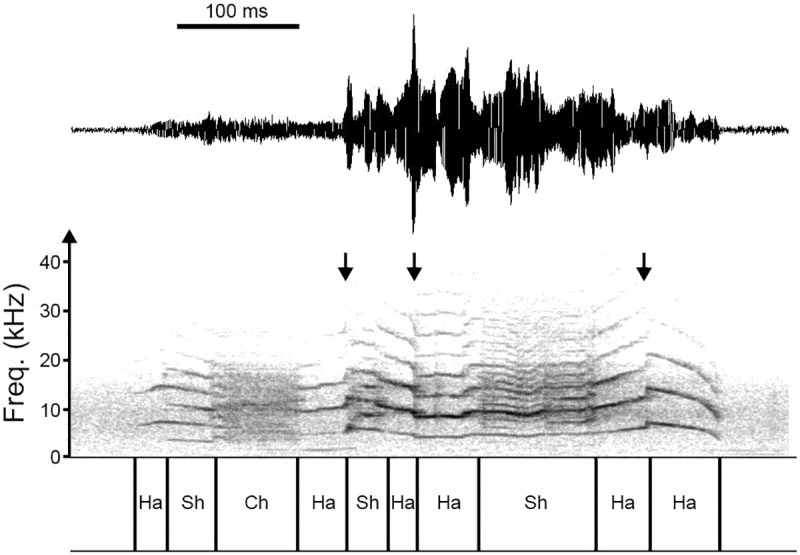
Temporal segmentation of calls. Shown here is the waveform (top trace) and spectrogram (bottom trace) of a call of female *Odorrana tormota*. The call was segmented into segments containing harmonic (Ha), subharmonics (Sh), deterministic chaos (Ch). This particular call does not show biphonation segments. Downward arrows indicate the times of occurrence of frequency jumps (FJ).

Calls were initially analyzed using SELENA, a custom-designed software [1,3–4] to display their narrow-band spectrograms and determine the call duration. For calls comprising multiple notes, we determined the durations of individual notes, the durations of signal breaks, as well as the overall call duration. For each call, we then employed PRAAT [21–22] to segment the call into different temporal segments, based on visual inspection of its narrowband spectrogram (Fig 1). Segment borders were placed at bifurcations, i.e., at boundaries between different dynamic regimes. The categories of different dynamic regimes were: no phonation, harmonic phonation, deterministic chaos, biphonation, subharmonics, and signal break [4,23–25]. Subharmonics existed at 0.5, 0.33, or 0.25 of the call fundamental frequency (*f*_0_). A frequency jump is the bifurcation between two harmonic segments with different *f*_0_s. Following call segmentation, we determined the time and frequency of occurrence of each segment, and measured the durations of the various segments (i.e., harmonic, subharmonic, deterministic chaos, biphonation). We additionally calculated these durations as percentages of the total call duration.

For each harmonic segment, we tracked the *f*_0_ using the “pitch tracking” mode in PRAAT (v.5.4.13) with 1-ms intervals. We did not measure the *f*_0_ for all nonlinear segments. Thus, for calls containing NLP, the *f*_0_ values represented the *f*_0_ in their harmonic segments only. During *f*_0_ tracking, we inspected the *f*_0_ track after it was overlaid on the call’s narrowband spectrogram. If the *f*_0_ track deviated from the first harmonic, the *f*_0_ track was recomputed using different thresholds and octave cost values in PRAAT, until the deviation was completely eliminated. With tracking of *f*_0_, we measured the mean *f*_0_ (*f*_0_Mean), maximum *f*_0_ (*f*_0_Max), minimum *f*_0_ (*f*_0_Min), and the difference between the maximum and minimum *f*_0_ (*f*_0_Max—*f*_0_Min). Furthermore, for each call we measured six temporal parameters: duration of harmonic segments (Ha duration), duration of frequency jump segments (FJ duration), duration of subharmonics segments (Sh duration), duration of biphonation segments (BP duration), duration of deterministic chaos segments (Ch duration), and duration of signal break (Brk duration).

To quantify call variability, for each sound parameter we calculated the between- and within-individual coefficients of variation, using the formula: CV = 100 X (Standard Deviation/Mean) [4, 9]. For within-female coefficients of variation (CV_w_), we used the means and standard deviations calculated from the calls of each female. For between-female coefficients of variation (CV_b_) we used the grand mean and standard deviation. We additionally computed the ratio of between- and within-female coefficients of variation (CV_b_/CV_w_) as a measure of relative variability among individuals. Parameters having a CV_b_/CV_w_ >1.0 indicate that they are relatively more variable among, than within, individuals and thus may potentially serve as a recognition cue [26,27]. Within-individual variability can be attributed to many factors, including the ambient temperature [28] and noise level. As recordings were made within narrow ranges of ambient temperature (16–19°C) and noise level (38–43 dB SPL RMS) indoors, much of the variability represented the differences in frog’s neuromuscular control of the vocal apparati at different times.

### Statistical analysis

We employed Shapiro–Wilk test to determine whether the data for each sound parameter were normally distributed, i.e., p > 0.05. As the data for all sound parameters were normally distributed, to determine whether or not vocalizations of individual females are distinct and contain individual vocal signatures, we carried out three different analyses, following the methods adopted in an earlier study in males *O*. *tormota* [4]. We first performed multivariate ANOVA to determine whether vocal signals from different individuals were significantly different; for post hoc comparisons we used Tukey’s HSD tests. For this, we included only the data collected from 5 females in 2015, excluding the data collected from two females in 2013 to avoid the possibility of repeat sampling. We next performed univariate ANOVA to determine whether and which of the 10 sound parameters were significantly different between individuals. Lastly, we carried out a stepwise discriminant function analysis (DFA) to predict group membership for each call. The result was a percentage documenting the correct assignment to individuals. To keep the sample size more uniform, we took the first 20 vocal signals from each of the five females, thus a total of 100 calls. Finally, for external validation, we additionally employed a leave-one-out cross validation procedure [29]. These analyses were carried out using SPSS v.17.0 (IBM Corp.).

The a priori probability of correct assignment by the DFA was based on the total number of groups. Thus, the probability of a call belonging to 1 of 5 females was one-fifth of 100%, or 20%. But, this procedure did not take into account the characteristics of the specific data [30]. Therefore, we calculated an alternative chance level on the basis of a randomized version of the actual data set (see http://www.random.org for a random sequence of the numbers from 1 to 100, using “sequence generator” function provided–this sequence was linked to the original data set). The number of calls per female was unchanged. The randomized set was then subjected to DFA, resulting in an average correct assignment.

## Results

We recorded 671 calls from 6 female *O*. *tormota*. All vocalizations were recorded from females in the gravid stage only, when their bellies were bloated and filled with eggs. We did make conscious efforts to record from 28 non-gravid females over the course of two reproductive seasons (2013 and 2015), but not once were we able to obtain a single recording of the vocalization of these females.

**Fig 2:**
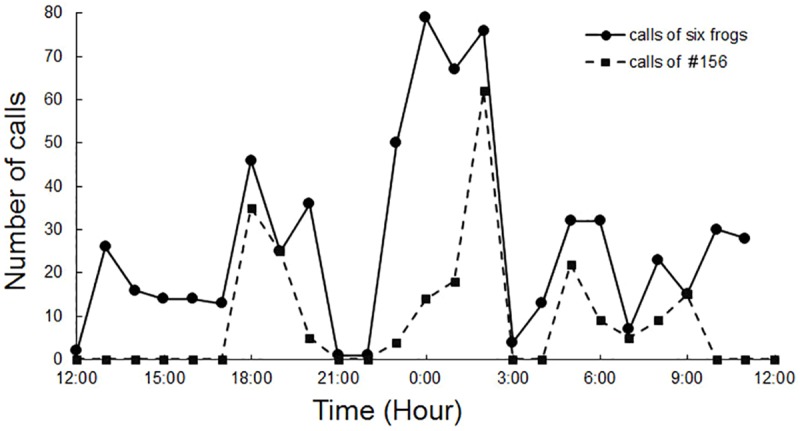
Time of calling of female *O*. *tormota*. Temporal distribution of calling activities of female *Odorrana tormota*. Shown are the composite data from six females (solid line; N = 671 calls), as well as the data from one of the females (dashed line; N = 174 calls).

Most captive gravid females vocalized throughout the day and night, in bouts, with peak calling periods at dusk (18:00–20:00 hours) and around midnight (23:00–02:00 hours) (Fig 2). At its peak, the calling rate reached 10 calls per minute. One gravid female in 2013, however, did not emit any call in captivity, much like the non-gravid females studied.

**Fig 3:**
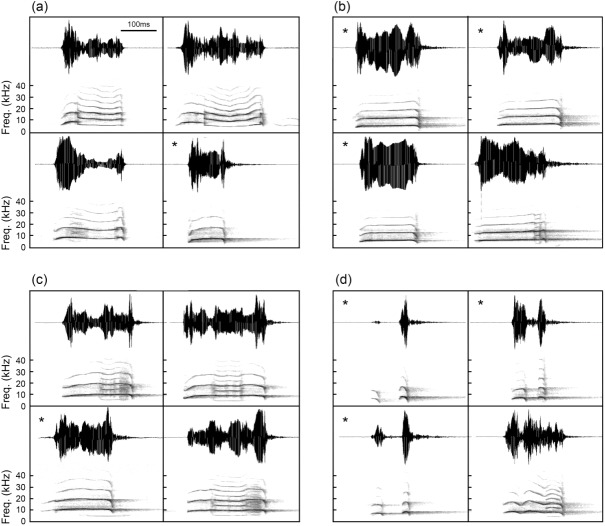
Calls of female *O*. *tormota* show individual variability. Representative calls of four females *Odorrana tormota*. Shown are four calls from each female (**a** through **d**). The majority of calls of two females (Female #152 in **a** and Female #155 in **c**) contain subharmonics, deterministic chaos, and/or frequency jumps (see Fig 1 legend for definitions of these terms). In contrast, most of the calls of the other two females (Female #154 in **b** and Female #156 in **d**) show little or no trace of NLP. Calls not exhibiting NLP are marked with *.

Most female vocal signals exhibited pronounced and complex upward/downward frequency modulations (Fig 1, S1 File). Their calls were diverse as exemplified by the representative calls of four females (Fig 3). Notably, for two of the females, the majority of their vocalizations contained prominent NLP (Fig 3A and 3C), with some segments of a call showing subharmonics, others showing deterministic chaos and/or frequency jump, while the remainders showing linear harmonic segments (see Fig 1 for explanations of these terms). In contrast, for the other two females (Fig 3B and 3D), the majority of their calls contained harmonic segments only, with no evidence of NLP. These results revealed that vocalizations of female *O*. *tormota* were diverse and variable, sometime their calls contained prominent NLP, other times their calls showed little or no evidence of NLP.

**Fig 4:**
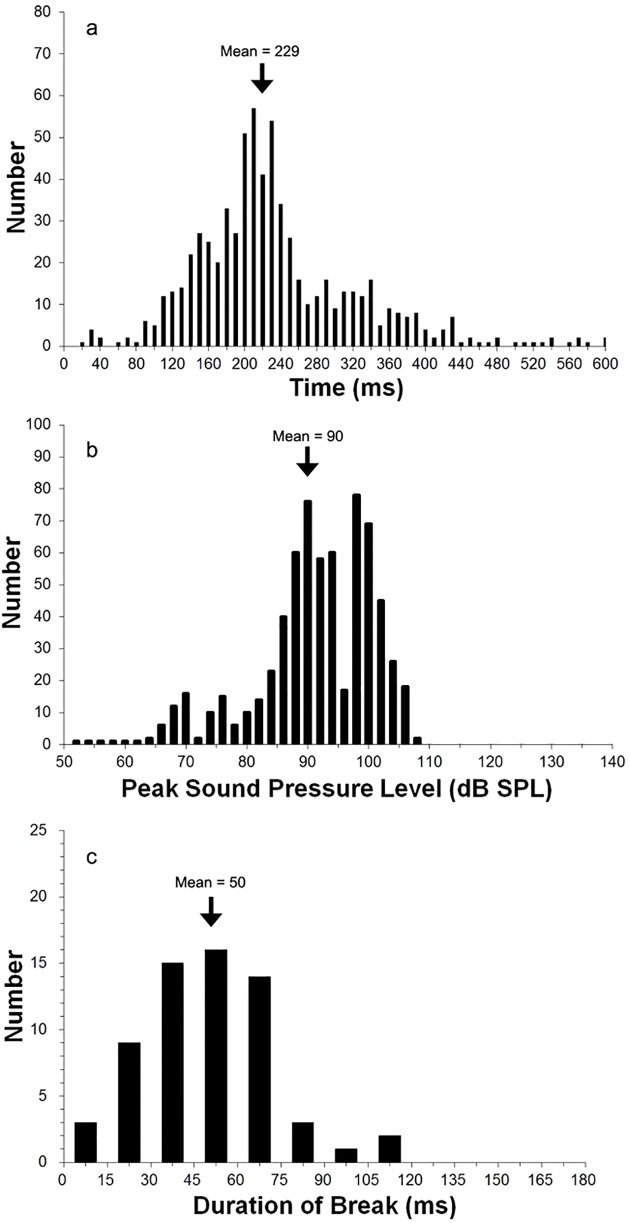
Temporal and intensity characteristics of calls of female *O*. *tormota*. (a) Distribution of the call duration of female *Odorrana tormota*. Shown is the data from six females (N = 671 calls); note that there are five overflow data points beyond the 600 ms range. (b) Distribution of the call intensity (N = 671 calls). (c) Distribution of the duration of signal break in multi-note calls (N = 63 calls).

The majority of the vocal signals of female *O*. *tormota* comprised single notes with a duration averaging 230.0 ± 94.1 ms (mean and standard deviation; Fig 4A)–this was shorter than the duration of males’ advertisement calls (namely, their long calls), whose averages range from 260 to 490 ms [4]. For two females, however, most of their calls consisted of multi-note calls (i.e., two or three successive notes), with signal breaks ranging between 11 and 115 ms, with a mean of 50.0 ± 22.6 ms (Fig 4B).

The call intensity of female vocalizations ranged widely. To assess the relative intensity of their calls, we measured the peak sound pressure level (SPL) of each call, a parameter that is essentially independent of the call duration. The peak SPL ranged from a low of 50 dB SPL to a high of 107 dB SPL, with a mean of 90.4 ± 9.7 dB SPL and a skewed distribution toward the higher end of the range (Fig 4C).

Of the 671 vocal signals recorded (S2 File), 264 (39%) displayed at least one of the NLP; the rest were without NLP. Of the 264 calls exhibiting NLP, 206 had at least 1 subharmonics segment, 191 displayed at least 1 frequency jumps, 77 showed more than 1 biphonation segments, 77 contained at least 1 deterministic chaotic segment. As noted above, there were marked differences in the frequency of occurrence of NLP from one female to the next (Table 1), some female emitted calls with low incidence of NLP (e.g., Female #134), others produced calls containing high incidence of NLP (e.g., Female #155). The relative duration of each of the different NLP segments varied from one female to another (Table 2).

**Table 1:** Frequency of occurrence of NLP in vocalizations of female *O*. *tormota*.

**Frog ID**	N	Pure Ha	Pure Ha (%)	FJ	FJ (%)	Sh	Sh (%)	BP	BP (%)	Ch	Ch (%)
**#134**	26	25	96.2	0	0	1	3.8	0	0	0	0
**#151**	29	8	27.6	19	65.5	19	65.5	2	6.9	0	0
**#152**	116	46	39.7	61	52.6	57	49.1	20	17.2	5	4.3
**#154**	103	77	75	20	19.4	26	25.2	2	0.02	0	0
**#155**	222	85	38.3	81	35.7	98	43.2	49	21.6	66	39.8
**#156**	175	166	94.9	10	5.7	5	2.9	4	2.2	0	0

The table above shows the frequency of occurrence of various NLP segments in vocalizations of female *Odorrana tormota*. Abbreviations: **Ha** represents harmonic segment; **FJ** represents frequency jump segments; **Sh** represents subharmonics segments; **BP** represents biphonation segments; **Ch** represents deterministic chaos segments.

**Table 2:** Temporal characteristics of calls of female *O*. *tormota*.

Frog ID	N	Duration(s)	Ha duration (%)	Sh duration (%)	BP duration (%)	Ch duration (%)	BrK duration (%)
**#134**	26	0.18 ± 0.075	87.6 ± 18.96	0.35 ± 1.79	0 ± 0	0 ± 0	12.08 ± 19.11
**#151**	29	0.29 ± 0.07	90.3 ± 10.9	8.9 ± 11.1	0.36 ± 1.36	0 ± 0	0.6 ± 2.26
**#152**	116	0.19 ± 0.057	88.5 ± 13.9	8.9 ± 13.9	1.4 ± 5.7	0.37 ± 2.1	0.78 ± 4.93
**#154**	103	0.22 ± 0.03	97.7 ± 7.85	1.9 ± 4.7	0.04 ± 0.27	0 ± 0	0.38 ± 3.9
**#155**	222	0.299 ± 0.11	80.54 ± 22.6	12.2 ± 18.3	1.43 ± 3.3	4.3 ± 3.3	1.3 ± 5.99
**#156**	175	0.17 ± 0.04	95.9 ± 12.9	0.46 ± 3.14	0.08 ± 0.56	0 ± 0	3.54 ± 12.4

This table shows the call duration and relative durations (mean ± standard deviation) of the harmonic segments, the various NLP segments, and the signal break in the calls of female *Odorrana tormota*. Abbreviations: **Ha** represents harmonic segment; **FJ** represents frequency jump segments; **Sh** represents subharmonics segments; **BP** represents biphonation segments; **Ch** represents deterministic chaos segments; **Brk** represents signal break.

In addition to individual differences in the frequency of occurrence of NLP, the spectral characteristics of the calls, such as the *f*_0_Mean, *f*_0_Max, *f*_0_Min, and *f*_0_Max–*f*_0_Min, differed from one female to the next (Table 3). The *f*_0_Mean ranged from a low of 6.5 kHz to a high of 9.3 kHz–*f*_0_Mean did not seem to relate to the frog’s snout-vent length (R^2^ = 0.15, p = 0.42). The grand *f*_0_Mean was 7.3 ± 0.9 kHz–this was not statistically different (two tailed t-test, p = 0.889) from the *f*_0_Mean (7.75 ± 0.92 kHz) reported for the males in the same study site [20]. The *f*_0_Max ranged from 7.0 kHz to 10.0 kHz, whereas *f*_0_Min ranged from 4.7 kHz to 7.4 kHz. Linear aggression analyses showed that, in addition to the *f*_0_Mean described above, the remaining sound parameters also did not show a correlation with the frog’s body length (p > 0.05).

**Table 3:** Spectral characteristics of calls of female *O*. *tormota*.

Frog ID (SVL)	N	*f*_0_Mean (kHz)	*f*_0_Max (kHz)	*f*_0_Min (kHz)	*f*_0_Max–*f*_0_Min (kHz)
**#134 (5.2)**	26	9.3 ± 0.7	9.9 ± 6.6	7.4 ± 1.1	2.5 ± 9.3
**#151 (6.4)**	29	8.0 ± 5.1	10.0 ± 5.4	6.4 ± 9.9	3.7 ± 1.0
**#152 (6.3)**	116	7.4 ± 7.7	8.9 ± 7.1	5.4 ± 7.1	3.5 ± 8.0
**#154 (5.8)**	103	6.5 ± 1.8	7.0 ± 3.6	4.7 ± 7.1	2.3 ± 6.1
**#155 (5.7)**	222	7.0 ± 9.5	7.9 ± 8.0	5.3 ± 1.2	2.6 ± 9.4
**#156 (4.8)**	175	7.7 ± 5.0	8.4 ± 4.2	5.4 ± 7.5	2.9 ± 7.4

**This table shows a q**uantitative description of the spectral characteristics of the calls of female *Odorrana tormota*. Shown are the average values and standard deviations of the call fundamental frequency (*f*_0_), the maxim *f*_0_, the minimum *f*_0_, and the difference between the maximum and minimum *f*_0_. SVL represents the frog’s snout-vent length and is shown in cm.

To quantify the variability in calls and assess how inter-individual variability compared to intra-individual variability, we measured the between- (CV_b_) and within-individual (CV_w_) coefficients of variations of ten acoustic parameters for the full data set (N = 671) from six females. These data are shown in Table 4. We noted that, for most sound parameters (except the durations of subharmonics, and deterministic chaos and signal break segments), the CV_w_ approximated the CV_b_, with the ratio CV_b_/CV_w_ approaching 1. However, the ratio for duration of deterministic chaos was far above 1, indicating that it varied far greater between individuals than within individuals, and thus it might be individual specific. Also, the CV_w_ for the *f*_0_Mean and *f*_0_Max were particularly small (i.e., <10), suggesting that these parameters were least variable and therefore might be individual specific. Similarly, the CV_w_ for the total duration, the duration of harmonic segments, and the *f*_0_Min and *f*_0_Max–*f*_0_Min were also relatively small (i.e., < 33), and these parameters too might be individual specific.

**Table 4:** Variability of temporal and spectral characteristics of calls of female *O*. *tormota*.

Parameter	Mean CV_w_ (Range)	Mean CV _b_	CV _b_ ⁄ CV_w_
**Total duration**	28.3 ± 10.2 (12.0–40.2)	28.5	1.01
**Duration of Ha**	33.3 ± 12.6 (14.4–50.6)	32.7	0.98
**Duration of Sh**	311.9 ± 236.2 (128.4–698.4)	159.2	0.51
**Duration of BP**	414.3 ± 264.6 (0–716.3)	368.4	0.89
**Duration of Ch**	128.1 ± 230.4 (0–569.4)	213.4	1.67
**Duration of Brk**	493.1 ± 290.9 (158.6–1014.9)	268.1	0.54
***f***_**0**_**Mean**	7.7 ± 3.7 (2.7–13.4)	7.7	1
***f***_**0**_**Max**	6.3 ± 2.0 (5.0–10.1)	6.2	0.99
***f***_**0**_**Min**	15.6 ± 3.2 (13.1–21.8)	15.4	0.99
***f***_**0**_**Max—*f***_**0**_**Min**	28.6 ± 5.2 (23.0–36.7)	28.3	0.99

This table shows the coefficients of variations of ten sound parameters (including call duration and durations of various harmonic and NLP segments) of 671 calls from six females.

To assess whether vocalizations of female *O*. *tormota* were individual specific and contained individual signatures, we first performed multivariate ANOVA of 100 calls from five females. For this, we took the first 20 calls recorded from each of the five frogs, all recorded at a background noise level of 38–43 dB SPL. The ANOVA test revealed significant differences between calls from individual females (Wilk's λ = 0.001, F = 29.90, p < 0.001). Subsequent univariate ANOVA tests showed that all 10 sound parameters, along with the relative values of NLP, were significantly different between individuals (Table 5).

**Table 5:** Results of univariate ANOVA tests that examined between-female variability.

Acoustic variable	F	P
**Total duration (s)**	42.7	< 0.001
**Harmonic segment duration (s)**	32.2	< 0.001
**Subharmonic segment duration (s)**	7.1	< 0.001
**Biphonic segments duration (s)**	46.0	< 0.001
**Chaotic segments duration (s)**	3.0	0.021
**Break duration (s)**	8.9	< 0.001
***f***_**0**_**Mean**	46.0	< 0.001
***f***_**0**_**Max**	323.2	< 0.001
***f***_**0**_**Min**	113.5	< 0.001
***f***_**0**_**Max–*f***_**0**_**Min**	58.4	< 0.001
**Harmonic segment duration (%)**	4.1	0.004
**Subharmonic segment duration (%)**	5.9	< 0.001
**Biphonic segments duration (%)**	61.9	< 0.001
**Chaotic segments duration (%)**	2.8	0.030
**Break duration (%)**	9.8	< 0.001

This table shows the results of Model-II ANOVAs that examined between-female variability (N = 100, df = 4). The tests revealed that all 10 absolute sound parameters as well as their relative values were significantly different between females.

We next employed a stepwise forward discriminant function analysis (DFA) to identify the call parameters that contributed to call discrimination (S3 File). For this, we removed several parameters from the data set, leaving a subset of six uncorrelated parameters (total call duration, duration of subharmonics, duration of deterministic chaos, duration of signal break, *f*_0_Mean, and *f*_0_Min). The analysis (Table 6) showed that the average correct assignment of the original data set was 87%. Namely, on average, 87% of the calls were correctly classified to five individuals. Application of leave-one-out cross validation DFA procedure yielded a slightly higher value (92.5%). In contrast, the average correct assignment of the randomized samples used to determine the chance level of correct assignment was much lower, ranging from 14% to 20%.

**Table 6:** Results of stepwise forward discriminant function analysis (DFA).

Frog ID	N	Original data set	Random data set #1	Random data set #2	Random data set #3	Random data set #4	Random data set #5
**#151**	20	80	20	10	20	20	20
**#152**	20	65	15	15	25	25	10
**#154**	20	100	20	20	20	15	15
**#155**	20	100	15	15	15	15	25
**#156**	20	90	20	15	25	15	20
**Mean correct assignment (%)**		87	17	14	20	19	19
**One-sided paired t-test**			t = 10.78	t = 11.92	t = 8.28	t = 8.05	t = 12.93
			p<0.001	p<0.001	p<0.001	p<0.001	p<0.001

This table shows the results of discriminant function analysis. The results are based on correct classification of 100 calls from five female *Odorrana tormota*, based on six sound parameters. Shown is the correct classification for the original data set as well as of the randomized data set (#1 - #5). The one-sided *t* -test compares the correct classifications of the original data set with the respective random data sets. Significance level p < 0.05.

**Fig 5:**
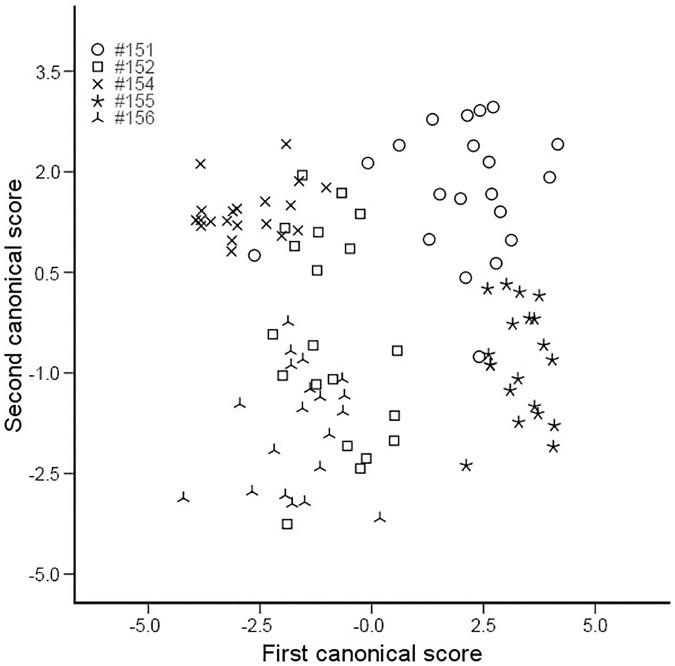
2D signal space plot of calls of female *O*. *tormota*. Shown here is the two-dimensional signal space plot of vocal calls of five individual females as defined by the first two canonical scores.

Fig 5 shows a plot of female calls in a two-dimensional signal space defined by the first two discriminant functions (or canonical scores). The figure revealed that although there were some overlaps among individual females, as to be expected due to the low CV_b_/CV_w_, the overlaps were minimal allowing the above-chance assignment of the DFA.

## Discussion

Results of the present study revealed that vocalizations of female *O*. *tormota* often exhibited pronounced and complex upward/downward frequency modulations and were diverse, and 39% of their calls contained at least one component of the NLP. This result validates our first working hypothesis. The present study represents the first to show evidence of NLP in vocal signals of female anurans. This discovery is at odds with the negative result reported previously in a study that focused on the acuity of sound localization of male *O*. *tormota* [5]. The discrepancy is presumably attributed to the small sample size in the earlier study, but is also in part due to the large inter-individual variability in call characteristics that we uncovered herein. We have found that some females emit calls containing little or no NLP, but other females produce calls most of which showing prominent NLP. Thus, results deriving from small samples are not generalizable to represent the population data.

An additional differential result reinforces the point above. The present study showed that the intensity of female vocalizations ranged widely, from 50 to 107 dB SPL; whereas some calls were of low and moderate intensity as reported previously [5], many female calls were as intense as those of male calls [3–4]. The high intensity and complexity of female *O*. *tormota* vocalizations appear to be unique among anuran amphibians. Generally, vocalizations of female anurans tend to be simpler and softer than male advertisement calls [12–13,15–19,31].

Another discrepancy between the present study and that of Shen and colleagues [5] lies in the call rate. Shen et al. reported that females call sparingly, at a rate of about one call per hour. We have found that females vocalize throughout the day, at least in captivity in the absence of males, and the calling rate fluctuates a great deal (Fig 2). At its highest rate, a female may emit up to 10 calls in a minute. Frogs’ calling rate is dependent on numerous factors, ranging from the frog’s physiological condition to various external factors such as the ambient temperature and the ambient noise level [32]. Our field observations reveal that females do not call when: 1. they are not gravid, 2. the water level is very high leading to ambient noise level of >70 dB SPL RMS, 3. the ambient temperature is below 15°C. Because our sound recordings were carried out indoors where the environmental factors were nearly constant, the variability and fluctuation in calling rate are likely attributable to differences and changes in the frog’s physiological condition.

The proportion of female calls containing NLP (39%) is lower than that for male *O*. *tormota*, for whom 93% of vocal signals exhibit NLP [4]. This sexual difference in calls is likely due to the anatomical differences in the vocal folds of the two sexes. In males, the vocal folds are complex and highly non-homogeneous dorso-ventrally; female’s vocal folds are also complex but not as non-homogeneous when compared to those of males [6]. Whether or not the variability in the anatomy of the vocal folds in the two sexes alone can account for the differential NLP occurrence requires experimental verification, however.

Discriminant function analysis of calls of female *O*. *tormota* showed that their calls are discriminable with a high likelihood. Stepwise DFA can correctly classify 87% of the calls to five individuals (Table 6). This classification level is comparable to that of male bullfrogs (92% for five males; [8]), male green frogs (52–100%; [9]) and male Aromobatid frog (65–94%; [11]), and comparatively higher than that observed in male *O*. *tormota* (55%; [4]).

Differences in the calls of female *O*. *tormota* occur in the temporal and spectral characteristics of the calls (the call duration and duration of signal break, and *f*_0_Mean, *f*_0_Max, *f*_0_Min, and *f*_0_Max–*f*_0_Min, as well as in the frequency of occurrence of NLP (Table 5). As suggested previously [24–25,33], the occurrence of NLP in vocal signals contributes to individual specificity. Our results support the above tenet as we found that the durations of subharmonics, deterministic chaos, biphonation and frequency jump all contribute to signal discrimination among females, despite the very large CV_w_ and low CV_b_/CV_w_ (Table 4). Taken together, results of the present study validate the second working hypothesis, namely, that female calls contain individual-specific characteristics. The differences in individual vocalizations seem to be uncorrelated with their differential body sizes–this conclusion, however, must be considered tentative in light of the limited sample size.

The individuality in sound communication signals of female *O*. *tormota* likely confers selective advantages. At this time, the behavioral advantages of individual specificity in female vocal signals are unclear. Male *O*. *tormota* have been shown to possess the ability to discriminate strangers from neighbors [7] in light of the individual signatures in their vocalizations [4]. Given the individual specificity of female vocalizations, it is possible that male *O*. *tormota* may also discriminate individual females acoustically. Likewise, assuming that females possess similar sound processing ability as do their male counterparts, it is also possible that female *O*. *tormota* may discriminate individuals in both sexes acoustically. However, further research, perhaps involving playback experiments [27], is necessary to determine whether or not males and/or females possess this discrimination ability.

What functions do female vocalization serve? Our current understanding of this issue is limited and incomplete, in part because female vocalization is uncommon among anurans. Various studies reporting female vocalizations have suggested that their vocal signals may serve to advertise receptivity [5,16,34], attract mate [5,18,35–36], select dominant mate through promotion of male-male competition [31], find or localize mate [18–19,35,37] promote courtship and formation of amplexus [13,34,38], or is involved in female-female competition [34]. That female *O*. *tormota* vocalize only when they are gravid in the peak of their reproductive season (as reported also for other species above) suggests that their vocalizations likely play a role in sexual advertisement, promotion of courtship and/or mate attraction. Indeed, their vocalizations have been shown to evoke male’s phonotaxis [5]. Interestingly, gravid females themselves are also attracted to the advertisement calls of male *O*. *tormota* [39]. At this time, it is unclear how males and females *O*. *tormota* interact in the wild acoustically or behaviorally prior to formation of amplexus. We do not know whether amplexus is the simple culmination of female’s phonotaxis towards a calling male–a strategy commonly used in many anurans species [32,40], or of male’s phonotaxis toward a calling female, or neither, or both but also involving additional acoustic and other behavioral interactions. Because female’s vocalizations can be as intense as male’s advertisement call (Fig 4B), in the wild they likely would elicit many males to respond phonotactically. The end result may be a massive scramble, with not easily predictable outcome for amplexus pairing. Clearly, further investigation is needed to understand more fully the instances, the purposes, and the targeted receiver of female vocalization in *O*. *tormota*, and in anuran generally.

## Supporting information

S1 File Vocalizations of a female #152. Shown here are audio files (N = 116) of vocalizations of one female (#152).
(RAR)

S2 File Data set of analyzed female vocal signals. Shown here is the full Excel data set of analyzed vocal calls.
(XLSX)

S3 File Data set used in discriminant function analysis. Shown here is the partial Excel data set used in discriminant function analysis.
(XLS)
